# Food Insecurity and Food Addiction in a Large, National Sample of Lower-Income Adults

**DOI:** 10.1016/j.cdnut.2023.102036

**Published:** 2023-11-20

**Authors:** Cindy W. Leung, Lindsey Parnarouskis, Melissa J. Slotnick, Ashley N. Gearhardt

**Affiliations:** 1 Department of Nutrition, Harvard T.H. Chan School of Public Health, Boston, MA, United States; 2 Department of Psychology, University of Michigan, Ann Arbor, MI, United States; 3 Department of Nutritional Sciences, School of Public Health, University of Michigan, Ann Arbor, MI, United States

**Keywords:** Food insecurity, food addiction, problematic eating behavior, disordered eating, ultra-processed foods

## Abstract

**Background:**

Growing research has highlighted associations between food insecurity and eating-related problems. Food addiction is one important, clinically significant pattern of problematic eating, which is related to, but distinct from, eating disorders. To date, there is only one study examining the association between food insecurity and food addiction, to our knowledge. Additional research is needed to understand the complexities of this association.

**Objective:**

We examined the association between food insecurity and food addiction in a large, national convenience sample of lower-income adults and potential heterogeneity in this association by age, gender, race, and ethnicity.

**Design:**

A cross-sectional, web-based study was conducted among 1780 US adults (≥18 y) with household incomes <250% of the federal poverty guideline. Household food security was assessed using the Household Food Security Survey Module. Food addiction was assessed using the modified Yale Food Addiction Scale (mYFAS), version 2.0. Multivariate logistic regression models examined the associations between food insecurity and food addiction, adjusting for sociodemographic covariates.

**Results:**

The prevalence of food addiction was 7.3%, and the prevalence of food insecurity was 51%. Compared with adults with food security, adults with food insecurity endorsed each mYFAS symptom with significantly greater frequency, including failure to fulfill major role obligations (20%), continued use despite social or interpersonal problems (18%), and craving or strong desire to use (16%). After adjustment, food insecurity was associated with 3.82-fold higher odds of food addiction (95% CI 2.36, 6.19), with no significant heterogeneity by age, gender, or race and ethnicity. The most problematic foods reported by adults with food insecurity were chips, nondiet soda, chocolate, pizza, and ice cream.

**Conclusion:**

These findings provide additional support for the association between food insecurity and food addiction. Mechanistic studies are needed to explore the role of psychosocial factors, eating behaviors, and the food environment in contributing to these associations.

## Introduction

Household food insecurity, a condition of inadequate access to nutritious food, remains a serious and persistent public health concern in the United States. In 2021, 10.2% of US households experienced food insecurity at some point over the past year, disproportionately affecting persons of minority race and ethnicity and those with greater socioeconomic disadvantage [1]. Food insecurity has known nutritional and health implications, including associations with poor diet quality, higher body mass indices, markers of systemic inflammation, hypertension, diabetes, and cardiovascular disease [[2], [3], [4], [5]]. More recently, an emerging area of research has focused on the study of food insecurity and eating-related problems with a specific focus on eating disorders (like anorexia, bulimia, and binge eating disorder) and associated disordered eating behaviors. These studies have found that dietary restraint, binge eating disorder, and unhealthy weight control behaviors are more prevalent among individuals with food insecurity [[6], [7], [8]].

Food addiction is one important and clinically significant pattern of problematic eating, which has been found to be related to, but distinct from, other types of disordered eating [9, 10]. Food addiction theorizes that highly processed foods with refined carbohydrates and/or added fats are sufficiently rewarding that they can trigger an addictive process in some individuals [11]. The concept of food addiction is mostly commonly measured by the Yale Food Addiction Scale (YFAS), which applies the diagnostic criteria for a substance use disorder (e.g., diminished control over intake, intense craving, continued use despite negative consequences, withdrawal, tolerance) and applies it to the intake of highly palatable foods (e.g., chocolate, baked goods, pizza, potato chips). The YFAS has been the subject of extensive psychometric validation in both adults and children [[12], [13], [14]], and higher food addiction scores are associated with poorer quality of life and obesity [[15], [16], [17]]. The most common source of foods with addictive potential is ultra-processed options (i.e., industrially made processed foods with ingredients unavailable to a home chef) [18, 19]. In addition to being high in rewarding ingredients (e.g., added sugar, fat), ultra-processed foods are also easily accessible, low cost, and heavily marketed [20, 21]. Evidence is growing that repeated exposure to highly rewarding, ultra-processed foods can lead to reward-related neural changes, which may contribute to the development of addictive patterns of eating [22].

Populations at risk of food insecurity are more likely to be exposed to ultra-processed foods with higher addictive potential in their local food environment, as these options tend to be more affordable, accessible, and more heavily advertised to low-income communities [23, 24]. To date, only 1 study has examined this relationship. In 2 independent samples, food insecurity was significantly associated with greater symptoms of food addiction in both samples of low-income pregnant females and mothers of preadolescent children [25]. Further research is needed to better understand this association.

This study aimed to examine the association between food insecurity and food addiction in a large and national convenience sample of lower-income adults. We also explored heterogeneity in this association by age, gender, race, and ethnicity. Based on known mechanisms and prior research [[26], [27], [28], [29], [30]], we hypothesized an overall positive association between food insecurity and food addiction, with potentially stronger associations observed among younger adults, females, and individuals from minority racial/ethnic groups.

## Methods

### Study population

In December 2022, we fielded a web-based (Qualtrics) survey to 2898 US adults (≥18 y) with household income levels <250% of the 2022 Federal Poverty Guidelines (FPG). The objective of the survey was presented to participants as: “…to understand factors that affect your diet and health.” Participant recruitment was facilitated through Cloud Research (formerly TurkPrime) using their Prime Panels platform. Prime Panels has been shown to provide high-quality data for academic research from respondents that are nationally representative with respect to several sociodemographic characteristics and is particularly useful for targeting hard-to-reach groups [31]. Participant recruitment and data collection occurred between 9 December and 22 December 2022. Of the 2898 US adults invited to complete the survey, individuals were excluded if they did not provide consent (*n =* 227), failed the attention checks (*n =* 231), did not live in the United States (*n =* 89), did not meet the income criteria (*n =* 531), or provided straight-lined responses for multiple sections (*n =* 22). Individuals were also excluded if they were missing data on food insecurity or food addiction (*n =* 18). The final analytic sample comprised 1780 adults from 49 states and the District of Columbia (Supplemental Figure 1). Participants were compensated directly through Prime Panels using preestablished rates. The study was considered exempt by the University of Michigan Institutional Review Board.

### Household food security

Household food security was assessed using the US Household Food Security Survey Module (HFSSM). The HFSSM is the most widely used measurement of food insecurity in the United States [32]. It consists of 18 questions that assess the perceptions and experiences of adults and children in the household related to not having enough money to purchase food over the past 12 mo. The questions range in severity, ranging from “worrying about food running out” to “not eating for a whole day.” If no children are present, the questions pertaining to children’s experiences in the household are omitted. Per USDA guidelines, food insecurity was defined as ≥3 affirmative responses.

### Food addiction

Food addiction was assessed using the modified YFAS, version 2.0 (mYFAS 2.0) [13]. The original YFAS 2.0 was developed to assess symptoms of addictive-like eating behaviors by adapting the diagnostic criteria for substance use disorder in the Diagnostic and Statistical Manual of Mental Disorders (DSM)-5 [12]. The mYFAS 2.0 is a shortened version of the YFAS 2.0 intended to reduce participant burden, which comprises 13 questions and assesses the presence of 11 substance use disorder symptoms according to the DSM-5. Each of the questions asks the respondent to focus on the certain foods that people tend to have “difficulty controlling,” with provided examples including sweets (e.g., ice cream, chocolate), starchy foods (e.g., white bread, rolls, pasta), salty snacks (e.g., chips, pretzels), fatty foods (e.g., steak, bacon, pizza), and sugary drinks. Frequency of symptoms over the past 12 mo is reported on an 8-point response scale, from “never” [1] to “every day” [8]. To meet the threshold for a mYFAS 2.0 symptom, participants must endorse a frequency threshold for that specific question. To meet the threshold for food addiction, participants must endorse ≥2 symptoms and meet the threshold for clinical significance. Clinical significance was defined as a response of “2-3 times a week” or >1 of 2 questions: “My eating behavior caused me a lot of distress” and “I had significant problems in my life because of food and eating.”

Following completion of the mYFAS 2.0, participants were asked which food items they experienced problems with over the past year. “Problems” was defined as “trouble cutting down on the food or losing control over how much of the food you eat.” We further specified that “problems” did not include insufficient intake or inaccessibility of that food. Participants were shown a list of 35 foods in random order, which included items from all major food groups and beverages and ranged from unprocessed to ultra-processed. The provided list of 35 foods was identical to what was asked in previous studies that aimed to investigate what foods are reported as being most addictive [18, 33]. Participants were instructed to “select all that apply” and were provided the option of writing in additional foods not on the list at the end.

### Study covariates

Sociodemographic covariates for the present study were chosen based on known associations with food insecurity and food addiction. They include age (18–29, 30–39, 40–49, 50–59, ≥60 y), gender (woman, man, gender nonbinary or nonconforming), race and ethnicity (non-Hispanic White or Middle Eastern/ North African, non-Hispanic Black, Hispanic, non-Hispanic Asian or Pacific Islander, or “Other” including American Indian or Alaskan Native and Multiracial or Multiethnic), educational attainment (high school degree or fewer years, some college, college graduate), and household income relative to national guidelines. Race and ethnicity were self-reported and considered structurally racialized categories encompassing potential experiences of discrimination and other social, economic, and environmental drivers of health disparities. Annual household income (before taxes) was self-reported via 15 categories in $10,000 to $15,000 increments. Percent of income relative to the 2022 FPG was calculated by taking self-reported income category midpoint and dividing by 100% FPG for their self-reported household size. %FPG was then categorized as <100% FPG, 100%–<200% FPG, ≥200% FPG for analysis. Study participants had complete (nonmissing) data on all sociodemographic covariates listed.

### Statistical analysis

First, we estimated descriptive statistics for sociodemographic variables and food addiction. Differences by food security status were examined using chi-squared tests. Next, we examined bivariate associations between each individual mYFAS symptoms (e.g., loss of control, craving, withdrawal) and food security status; differences were examined using chi-squared tests. Using multivariate logistic regression models, we then examined associations between food security status and individual food addiction symptoms, and then food addiction threshold. Models were adjusted for sociodemographic covariates. We further explored potential moderation in these associations by participant’s age, gender, and race and ethnicity through testing the significance of cross-product terms between food security status and each demographic covariate in separate models.

All statistical tests were 2-sided and significance was considered at *P<0.05*. Statistical analyses were performed using SAS 9.4 (SAS Institute, Cary, NC).

## Results

In the analytic sample, the prevalence of food insecurity was 51%. Sociodemographic characteristics stratified by food security status of the sample are shown in Table 1. Adults with food insecurity were more likely to be of younger age, identify as a woman or gender nonconforming, identify as nonHispanic Black or Hispanic, have fewer years of educational attainment, and have lower household income than adults with food security (*Ps<0.0001*). Overall, 7.3% of adults met the criteria for food addiction; however, food addiction was more prevalent among adults with food insecurity (11.7%) compared with adults with food security (2.5%) (*P<0.0001*).

**TABLE 1:** Characteristics of the study sample by food security status

	Overall (*n =* 1,780)		Food secure (*n =* 868)		Food insecure (*n =* 912)		*P*^a^
	*n*	%	*n*	%	*n*	%	
Age (y)							<0.0001
18–29	326	18.3	114	13.1	212	23.3	
30–39	351	19.7	136	15.7	215	23.6	
40–49	328	18.4	137	15.8	191	20.9	
50–59	306	17.2	150	17.3	156	17.1	
≥60	469	26.4	331	38.1	138	15.1	
Gender							<0.0001
Females	915	51.4	397	45.7	518	56.8	
Males	849	47.7	468	53.9	381	41.8	
Gender nonconforming	16	0.9	3	0.4	13	1.4	
Race and ethnicity							0.12
NonHispanic White or MENA	1226	68.9	621	71.5	605	66.3	
NonHispanic Black	274	15.4	124	14.3	150	16.5	
Hispanic	170	9.6	75	8.6	95	10.4	
Asian, Pacific Islander or Hawaiian	30	1.7	16	1.8	14	1.5	
Other racial/ethnic group^b^	80	4.5	32	3.7	48	5.3	
Educational attainment							<0.0001
High school or less	707	39.7	300	34.6	407	44.6	
Some college	540	30.3	267	30.8	273	29.9	
College graduate	533	29.9	301	34.7	232	25.4	
Income to FPG (%)							<0.0001
<100% FPG	615	34.6	248	28.6	367	40.2	
100–<200% FPG	787	44.2	394	45.4	393	43.1	
≥200% FPG	378	21.2	226	26.0	152	16.7	
Food addiction							<0.0001
No food addiction	1651	92.8	846	97.5	805	88.3	
Food addiction	129	7.3	22	2.5	107	11.7	

MENA, Middle Eastern or North African; FPG, federal poverty guideline

a Significance for differences by food insecurity determined by chi-squared tests

b Includes adults who self-identified as American Indian or Alaskan Native, Multiracial, Multiethnic, or “other” race or ethnic group not listed

Table 2 shows the associations in the individual mYFAS symptoms by food security status. Compared with adults with food security, adults with food insecurity endorsed each mYFAS symptom with significantly greater frequency (all *Ps<0.0001*). Among adults with food insecurity, the 3 most frequently endorsed symptoms were: 1) failure to fulfill major role obligations (19.9%), 2) continued use despite social or interpersonal problems (17.8%), and 3) craving or strong desire to use (16.2%). Among adults with food security, the 3 most frequently endorsed symptoms were: 1) persistent desire or repeated unsuccessful attempts to quit (6.2%), 2) continued use despite social or interpersonal problems (6.0%), 3) failure to fulfill major role obligations (5.3%). After adjusting for sociodemographic covariates, food insecurity remained significantly associated with elevated odds of each mYFAS symptom, ranging from substance consumed in larger amount and for longer period than intended (odds ratio [OR] 1.87, 95% confidence interval [CI] 1.21, 2.89) to failure to fulfill major role obligations (OR 3.44, 95% CI 2.42, 4.89).

**TABLE 2:** Associations between food insecurity and endorsement of individual food addiction symptoms

Symptom	Endorsement by overall sample (*n =* 1,780)		Endorsement by food secure adults (*n =* 868)		Endorsement by food insecure adults (*n =* 912)		Multivariate-adjusted associations with food insecurity^a^	
	*n*	%	*n*	%	*n*	%	OR	95% CI
Substance consumed in larger amount and for longer period than intended	111	6.2	33	3.8	78	8.6^b^	1.87	1.21, 2.89
Persistent desire or repeated unsuccessful attempts to quit	178	10.0	54	6.2	124	13.6^b^	2.08	1.46, 2.96
Much time/activity to obtain, use recover	133	7.5	40	4.6	93	10.2^b^	1.84	1.24, 2.74
Important social, occupational, or recreational activities given up or reduced	88	4.9	19	2.2	69	7.6^b^	2.78	1.64, 4.74
Use continues despite knowledge of adverse consequences	169	9.5	38	4.4	131	14.4^b^	2.87	1.95, 4.23
Tolerance (marked increase in amount, decrease in effect)	146	8.2	41	4.7	105	11.5^b^	2.16	1.46, 3.19
Characteristic withdrawal symptoms	168	9.4	45	5.2	123	13.5^b^	2.47	1.71, 3.57
Continued use despite social or interpersonal problems	214	12.0	52	6.0	162	17.8^b^	2.62	1.86, 3.69
Failure to fulfill major role obligations	227	12.8	46	5.3	181	19.9^b^	3.44	2.42, 4.89
Use in physically hazard situations	176	9.9	35	4.0	141	15.5^b^	3.40	2.29, 5.06
Craving or strong desire to use	192	10.8	44	5.1	148	16.2^b^	3.06	2.13, 4.41
Use causes clinically significant impairment or distress	166	9.3	34	3.9	132	14.5^b^	3.14	2.10, 4.69

a Multivariate logistic regression models adjusted for age, gender, race and ethnicity, educational attainment, and income (relative to federal poverty guideline)

b Significantly different from food secure group (*P<*0.0001) from chi-squared tests

Multivariate-adjusted associations between food insecurity and food addiction are shown in Table 3. In age- and gender-adjusted analyses, food insecurity was associated with 3.83-fold higher odds of food addiction (95% CI 2.38, 6.17). After adjusting for all sociodemographic covariates, the association between food insecurity and food addiction remained unchanged (OR 3.82, 95% CI 2.36, 6.19). There was no evidence of heterogeneity in the association between food insecurity and food addiction by participant’s age, gender, or race and ethnicity (all *Ps-interaction >0.2*).

**TABLE 3:** Associations between food insecurity and food addiction diagnostic criteria

	YFAS diagnostic criteria^a^			
	Age- and gender- adjusted		Multivariate-adjusted^b^	
	OR	95% CI	OR	95% CI
Food secure	Ref.	-	Ref.	-
Food insecure	3.83	2.38, 6.17	3.82	2.36, 6.19

a YFAS diagnostic criteria include endorsement of at least 2 individual symptoms and endorsement of “clinically significant impairment or distress”

b Multivariate logistic regression models adjusted for age, gender, race and ethnicity, educational attainment, and income (relative to federal poverty guideline)

Among adults with food insecurity, the top 5 problematic foods endorsed included: 1) chips (34.1%), 2) nondiet soda (33.0%), 3) chocolate (31.8%), 4) pizza (31.3%), and 5) ice cream (29.6%). Among adults with food security, the top 5 problematic foods endorsed included: 1) ice cream (24.7%), 2) chips (24.3%), 3) nondiet soda (22.4%), 4) chocolate (21.3%), and 5) cookies (20.7%) (data not shown).

## Discussion

In the current study of lower-income adults, we observed a strong overall association between food insecurity and food addiction. In fact, adults with food insecurity were 282% more likely to meet the clinical threshold for food addiction even when adjusting for sociodemographic correlates. The magnitude of this association is on par or exceeds the associations between food addiction and known addiction risk factors, including having a family history of addiction [34]. The association between food insecurity and food addiction was robust across age, gender, and race and ethnic groups. Furthermore, adults with food insecurity reported every mYFAS symptom with significantly higher prevalence than lower-income individuals with food security. Our findings are consistent with the previous study conducted in 2 independent samples of adult females, and suggest that this association between food insecurity and food addiction extends to the larger population of lower-income US adults, including males, adults of different ages, and more persons of minority racial and ethnic backgrounds [25]. As food addiction is associated with lower quality of life across every domain [15], it is important to screen individuals at risk for food insecurity for food addiction to inform the need for clinical care. Additionally, screening individuals presenting with food addiction for food insecurity may inform the need to ensure adequate access to nutritious food as an essential step in reducing compulsive intake of ultra-processed foods.

Although the cross-sectional nature of the study precludes causal inferences, there is mechanistic plausibility to support the association between food insecurity and food addiction. Food insecurity signifies an inability to access and afford nutritious food for one’s family due to financial hardship [35]. Dietary coping strategies for food insecurity can range from eating smaller meals and skipping meals entirely to food hoarding and binge eating [8, 35, 36]. Food insecurity is a chronic stressor that has been linked with serious psychological distress, anxiety, depression, and poor mental health [[37], [38], [39], [40]]. The stress of food insecurity may also compel individuals to seek out ultra-processed “comfort” foods to help mitigate their stress response [41]. These types of foods are ubiquitous in the food environment, and even more so in lower-income and minority racial/ethnic neighborhoods where food insecurity tends to be more concentrated [23, 24]. Food-insecure individuals have higher intakes of ultra-processed food, which can lead to hyperactivation of neural reward pathways and further reinforce intake [42]. The co-occurrence of these conditions—chronic stress, maladaptive dietary behaviors, and a pernicious food environment—suggest a pathway by which individuals with food insecurity may be at heightened risk for food addiction.

Although the most endorsed items among individuals with food insecurity and food security were similar—both included a failure to fulfill major role obligations (food insecure: 19.9% vs. food secure 5.3%) and continued use despite interpersonal consequences (food insecure: 17.8% vs. food secure: 6.0%), differences in symptom endorsement between these groups may warrant further study. Interestingly, the top 3 most endorsed items for individuals with food insecurity included craving (16.2%), whereas individuals with food security endorsed persistent desire or attempts to cut down (6.2%). Craving may be heightened among individuals with food insecurity by experiences of caloric and hedonic deprivation and increased food cues in food environment. Persistent attempts to cut down on ultra-processed foods may be more frequent in people with more flexibility around their diet and greater access to minimally processed foods. For example, if an individual must rely on ultra-processed foods to meet their caloric needs, attempting to cut down could mean choosing between eating and going hungry. Future research should consider these as starting points for examining potential pathways to explain the association between food insecurity and food addiction.

Adults in the present study across both food security groups endorsed foods such as chips, chocolate, nondiet soda, and ice cream as being problematic. This finding is consistent with other studies in more well-resourced samples that used a similar set of foods and identified the same types of food as most addictive [18, 33], reinforcing the theory that not all foods are equally likely to trigger addictive processes. Instead, ultra-processed foods containing high levels of refined carbohydrates and/or fats are theorized to have the highest addictive potential due to their ability to more intensely stimulate reward and motivation systems in the brain [19]. Further, high levels of both carbohydrates and fats are a rare occurrence in naturally occurring foods, but is extremely common in ultra-processed foods. There is evidence that this unnatural combination of carbohydrates and fats has a supra-additive effect in stimulating reward/motivation systems, further increasing the addictive nature of these foods [43]. In the current study, the top 5 rated as most problematic across both groups were entirely ultra-processed forms that include a combination of refined carbohydrates and fat, which supports the conceptualization of these foods as particularly addictive. Policy efforts are needed to target structural factors that increases exposure to and reliance on potentially addictive, ultra-processed foods, particularly in lower-income and food-insecure populations.

The primary limitation of the study is the cross-sectional nature of the data. Given food insecurity and food addiction were assessed simultaneously, it is not possible to determine the directionality of the association. Reverse causation may be plausible if severe food addiction leads to financial strain and subsequent food insecurity. Second, food insecurity coexists with poverty and other material insecurities. Interventions to address food addiction in populations at risk for food insecurity need to consider the broader socioeconomic, geographic, and policy contexts by which individuals fall into acute or chronic periods of food insecurity and identify opportunities to address these structural determinants. Another limitation is that the mYFAS 2.0 has not been psychometrically tested among individuals experiencing food insecurity. Those with food insecurity may have different interpretations to mYFAS 2.0 questions than those with food security, and our team is currently pursuing this research. Furthermore, our study did not include measures of clinical eating disorders or more broadly, disordered eating behaviors, though studies examining the relationships between food insecurity and eating disorders [[44], [45], [46]], and disordered eating [8, 36, 47] have previously been published. Future studies may want to examine disordered eating as a moderator of the relationship between food insecurity and food addiction.

Food insecurity was considered a dichotomous variable rather than the 4 established levels of food security (high, marginal, low, very low). Given that food addiction was relatively rare in our sample (7.3%), our sample was too small to consider all 4 categories of food security. Future studies with larger samples are needed to assess whether a dose-response relationship exists between food insecurity and food addiction. Another limitation is the inclusion of self-reported race and ethnicity as study covariates. Additional research is needed to understand the structural factors that drive food insecurity disparities in minoritized populations, including racial discrimination, employment and wage disparities, inequitable access to food stores, and more, which will better inform potential moderation of the association between food insecurity and food addiction within racial and ethnic groups. Finally, our study was conducted through CloudResearch using their Prime Panels platform. The online platform may have excluded those who are less tech savvy or without stable Internet connections, including individuals that might be at higher risk of food insecurity. Our study is strengthened by the large sample size, which provides sufficient power to examine the main association of interest and explore heterogeneity by important demographic factors. These characteristics support the generalizability of our findings, although additional research using different study methodology is needed to add to this emerging evidence base.

This study provides further support for the association between food insecurity and food addiction in a large and national convenience sample of lower-income adults. Although the associations between food insecurity, disordered eating, and eating disorder pathology have been established, food addiction is a distinct construct related to mechanisms implicated in substance addictions (e.g., reward dysfunction, withdrawal, intense cravings). Efforts to alleviate food insecurity and improve access to nutritious foods may also lead to favorable associations with dietary behaviors and food addiction symptomology.

## Author contributions

The authors’ responsibilities were as follows—CWL, LP, ANG designed research; MJS conducted research; CWL and MJS performed statistical analysis; CWL, LP, ANG wrote paper; CWL had primary responsibility for final content. All authors read and approved the final manuscript.

## Funding

This study was supported by a grant from the University of Michigan Office of Research.

## Data availability

Data described in the manuscript can be made available upon request.

## Conflict of interest

The authors have no conflicts of interest to disclose.
