# Supplementary material for: Food Insecurity and Food Addiction in a Large, National Sample of Lower-Income Adults
Source: Curr Dev Nutr. 2023 Nov 20;7(12):102036. doi: 10.1016/j.cdnut.2023.102036 (PMC10761353; doi:10.1016/j.cdnut.2023.102036)
Supplement: Multimedia component 1 [file mmc1.docx]

**Supplemental Figure 1: Analytic sample flow-chart**

All US adults ≥18 years with incomes <250% of the 2022 federal poverty guidelines who completed the survey

**n = 2,898**

**Final Analytic Sample = 1,780**

Incorrectly answered attention checks (n=231) or provided straight-lined responses for multiple sections (n=22)

Missing data on food insecurity or food addiction (n=18)

Did not live in the US (n=89) or meet the income criteria (n=531)

Did not provide consent (n=227)
